# Btk inhibitor ibrutinib reduces inflammatory myeloid cell responses in the lung during murine pneumococcal pneumonia

**DOI:** 10.1186/s10020-018-0069-7

**Published:** 2019-01-15

**Authors:** Alexander P. de Porto, Zhe Liu, Regina de Beer, Sandrine Florquin, Onno J. de Boer, Rudi W. Hendriks, Tom van der Poll, Alex F. de Vos

**Affiliations:** 1 Center for Experimental and Molecular Medicine (CEMM), Amsterdam UMC, Academic Medical Center, University of Amsterdam, Meibergdreef 9, Room G2-132, 1105 AZ Amsterdam, the Netherlands; 2 Amsterdam Infection and Immunity Institute (AI&II), Amsterdam UMC, Amsterdam, the Netherlands; 3 Department of Pathology, Amsterdam UMC, Academic Medical Center, University of Amsterdam, Amsterdam, The Netherlands; 4 Department of Pulmonary Medicine, Erasmus MC Rotterdam, Rotterdam, The Netherlands; 5 Division of Infectious Diseases, Amsterdam UMC, Academic Medical Center, University of Amsterdam, Amsterdam, The Netherlands

**Keywords:** Bruton’s tyrosine kinase, Ibrutinib, Pneumonia, *Streptococcus pneumoniae*, Sepsis

## Abstract

**Background:**

*Streptococcus pneumoniae* is a major causative agent in community-acquired pneumonia and sepsis. Overwhelming lung inflammation during pneumococcal pneumonia may hamper lung function. Ibrutinib is an irreversible inhibitor of Bruton’s tyrosine kinase (Btk), a key signaling protein controlling the activation of various immune cells, including macrophages and neutrophils. The aim of this study was to determine whether ibrutinib treatment ameliorates acute lung inflammation during pneumococcal pneumonia.

**Methods:**

Mice were treated orally with ibrutinib and the effect on acute pulmonary inflammation elicited by the gram-positive bacterial cell wall component lipoteichoic acid (LTA) and during ceftriaxone-treated pneumococcal pneumonia was assessed.

**Results:**

Treatment with ibrutinib prior to and after intranasal LTA instillation reduced alveolar macrophage activation, neutrophil influx, cytokine release and plasma leakage into the lung. Postponed treatment with ibrutinib supplementing antibiotic therapy during ongoing pneumococcal pneumonia did not impair bacterial killing in lung, blood and spleen. In this setting, ibrutinib reduced alveolar macrophage and systemic neutrophil activation and substantially diminished further monocyte and neutrophil influx in the lung. In vitro, ibrutinib inhibited macrophage TNF secretion and neutrophil activation upon LTA and pneumococcal stimulation.

**Conclusions:**

Taken together, these data indicate that the Btk inhibitor ibrutinib reduces inflammatory myeloid cell responses during acute pulmonary inflammation evoked by LTA and antibiotic-treated pneumococcal pneumonia and suggest that ibrutinib has the potential to inhibit ongoing lung inflammation in an acute infectious setting.

**Electronic supplementary material:**

The online version of this article (10.1186/s10020-018-0069-7) contains supplementary material, which is available to authorized users.

## Background

Respiratory infections by *Streptococcus (S.) pneumoniae* remain a major cause of community-acquired pneumonia and sepsis (Pneumoniae, n.d.; Musher & Thorner, 2014; Angus & van der Poll, 2013). The entry of pneumococci in the lower airways triggers a local innate host defense response, characterized by release of cytokines and chemokines leading to polymorphonuclear cell (PMN) and monocyte recruitment in order to expel the bacteria (van der Poll & Opal, 2009; Koppe et al., 2012). An exaggerated inflammatory response resulting from inadequate elimination of pneumococci, however, may hamper lung function (Thompson et al., 2017; van der Poll et al., 2017). Drugs that can reduce overwhelming lung inflammation during pneumonia may serve as adjuvant treatment in patients with community-acquired pneumonia (Muller-Redetzky et al., 2015).

Several pattern recognition receptors contribute to the initiation of an inflammatory response to pneumococci, such as Toll-like receptor 2 (TLR2), TLR4, TLR9, triggering receptor expressed on myeloid cells (TREM)-1 and inflammasomes (van der Poll & Opal, 2009; Koppe et al., 2012; Rabes et al., 2016; Hommes et al., 2014). Bruton’s tyrosine kinase (Btk) is a versatile signaling protein downstream several immune receptors (Weber et al., 2017), including TLR2, TLR4 and TLR9 (Liljeroos et al., 2007; Liu et al., 2011; Doyle et al., 2007), the Nod-like receptor family pyrin domain containing 3 (NLRP3) inflammasome (Ito et al., 2015) and TREM-1 (Ormsby et al., 2011). In macrophages, Btk regulates the secretion of inflammatory cytokines upon TLR, NLRP3 and TREM-1 stimulation (Weber et al., 2017; Liljeroos et al., 2007; Liu et al., 2011; Doyle et al., 2007; Ito et al., 2015; Ormsby et al., 2011). Furthermore, in PMN, Btk is involved in adherence to and crawling along the endothelium and migration into tissues (Yago et al., 2010; Mueller et al., 2010; Volmering et al., 2016). The fact that expression of Btk is confined to immune cells, including B cells and innate immune cells such as macrophages, monocytes and PMN (Weber et al., 2017), makes Btk an interesting target for intervention of the exaggerated inflammatory response during pneumococcal pneumonia.

Ibrutinib is an irreversible inhibitor of Btk, which inactivates its kinase domain and thereby the capacity of Btk to phosphorylate and activate downstream signaling proteins (Honigberg et al., 2010). Ibrutinib is currently used in patients for treatment of B cell malignancies (Hendriks et al., 2014) and chronic graft versus host disease (Miklos et al., 2017). Furthermore, prolonged treatment with ibrutinib has been found effective in chronic autoimmune disease models mediated by B cells (Crofford et al., 2016). Little is known, however, about the efficacy of ibrutinib in acute inflammatory responses in vivo mediated by myeloid cells (Florence et al., 2018). In the current study we investigated the potential of ibrutinib to inhibit acute inflammatory processes in the lung in mouse models of acute lung inflammation elicited by lipoteichoic acid (LTA), a pro-inflammatory component of the gram-positive cell wall, and antibiotic-treated pneumococcal pneumonia.

## Methods

### Animals

Btk^−/−^ and CD19-BTK mice were generated as previously described (Hendriks et al., 1996; Maas et al., 1999). C57Bl/6 male mice were bought from Charles River, Maastricht, Netherlands. All mouse lines were backcrossed at least eight times to C57Bl/6 background. All animals were specific pathogen-free and housed in the Animal Research Institute Amsterdam facility under standard care. All experiments were carried out in accordance with the Dutch Experiment on Animals Act and were approved by the local animal welfare committee of the Academic Medical Center.

### Experimental study design

Animals were 10 weeks of age at the start of experiments. Groups consisted of 8 mice unless mentioned otherwise. Mice were treated with vehicle or 25 mg/kg body weight ibrutinib (Selleckchem, Munich, Germany) in vehicle solution by oral gavage as previously described (Kil et al., 2012). Lung inflammation was induced by intranasal instillation of 100 μg LTA (Invivogen, San Diego, CA) as previously described (Hoogendijk et al., 2012). Ibrutinib and vehicle were administered 3 h prior to and 9 h after induction of lung inflammation. Pneumococcal pneumonia was evoked by intranasal inoculation with 2 × 10^5^ colony forming units (CFU) *S. pneumoniae* (serotype 3, strain 6303; ATCC, Manassas, VA) as described (Hoogendijk et al., 2012). Ibrutinib (25 mg/kg) and vehicle were given simultaneously with ceftriaxone (20 mg/kg, intraperitoneally; Fresenius Kabi, Zeist, the Netherlands), 24 h after infection. Vehicle or ibrutinib treatment was repeated at 36 h after induction of pneumonia. Bronchoalveolar lavage fluid (BALF), blood and organs were harvested and processed as described previously (Hoogendijk et al., 2012). Detailed experimental procedures are described in Additional file 1: supplemental methods.

### Cell stimulation and analysis of inflammatory responses

Cell stimulation, cell counting, flow cytometry, immunohistochemistry, pathology, measurement of organ damage, protein measurements, western blotting procedures and reagents can be found in the Additional file 1: supplemental methods.

### Statistical analysis

Statistical analysis was performed with GraphPad Prism software (San Diego, CA). Data are given as means and standard deviation (SD) in tables and bar graphs. Differences between groups were analyzed using Mann–Whitney U-test; comparisons between more than two groups were first performed using Kruskal-Wallis 1-way analysis of variance test. A value of *P* < 0.05 was considered statistically significant.

## Results

### Ibrutinib inhibits LTA and S. pneumoniae-induced macrophage and PMN activation in vitro

**Fig. 1:**
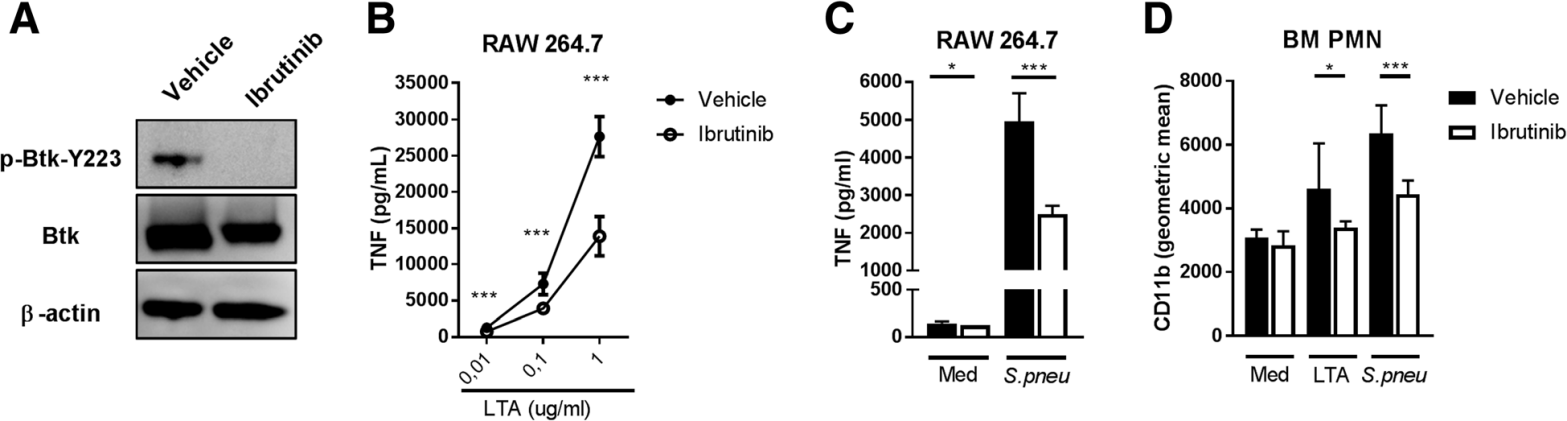
Ibrutinib inhibits LTA and *S. pneumoniae*-induced macrophage and PMN activation in vitro. **a** RAW264.7 macrophages were stimulated for 5 min with 10 μg/mL lipoteichoic acid (LTA) in the presence of 2500 nM Ibrutinib in IMDM containing 0,1% DMSO or vehicle (0.1% DMSO in IMDM). Cell lysates were analyzed by Western blot for total Bruton’s tyrosine kinase (Btk), Tyrosine (Y) 223 phosphorylated Btk and β-actin. **b** RAW 264.7 cells were stimulated with 0.01, 0.1 and 1 μg/mL LTA or (**c**) UV-killed *S. pneumoniae* strain 6303 (cell:bacterium ratio 1:100) (*n* = 8 wells group) for 24 h in the presence of 2500 nM Ibrutinib or vehicle. The concentration of tumor necrosis factor (TNF) in supernatant is depicted. **d** Bone marrow polymorphonuclear cells (PMN) were stimulated with LTA (10 μg/ml) or UV-killed *S. pneumoniae* strain 6303 (cell:bacterium ratio 1:100) (*n* = 8 wells group) for 1 h in the presence of 2500 nM Ibrutinib or vehicle. CD11b expression was determined by flow cytometry. Data are represented as the mean of each group with error bars representing SD. *p < 0.05, ***P < 0.001 (Mann–Whitney U-test). Data represent results from one (**d**), two (**a**) or three (**b** and **c**) independent experiments

Ibrutinib is an irreversible inhibitor of the kinase activity of Btk and able to inhibit autophosphorylation of Btk on tyrosine Y223 (Honigberg et al., 2010). Ibrutinib completely abrogated autophosphorylation of Btk Y223 in the murine macrophage cell line RAW264.7 stimulated with LTA (Fig. 1a) and reduced tumor necrosis factor (TNF) secretion upon stimulation with *S. pneumoniae* or different concentrations of LTA (Fig. 1b-c). Ibrutinib also significantly reduced LTA or *S.pneumoniae*-induced CD11b expression on PMN (Fig. 1d).

### Ibrutinib reduces LTA-induced myeloid cell responses during acute lung inflammation

**Fig. 2:**
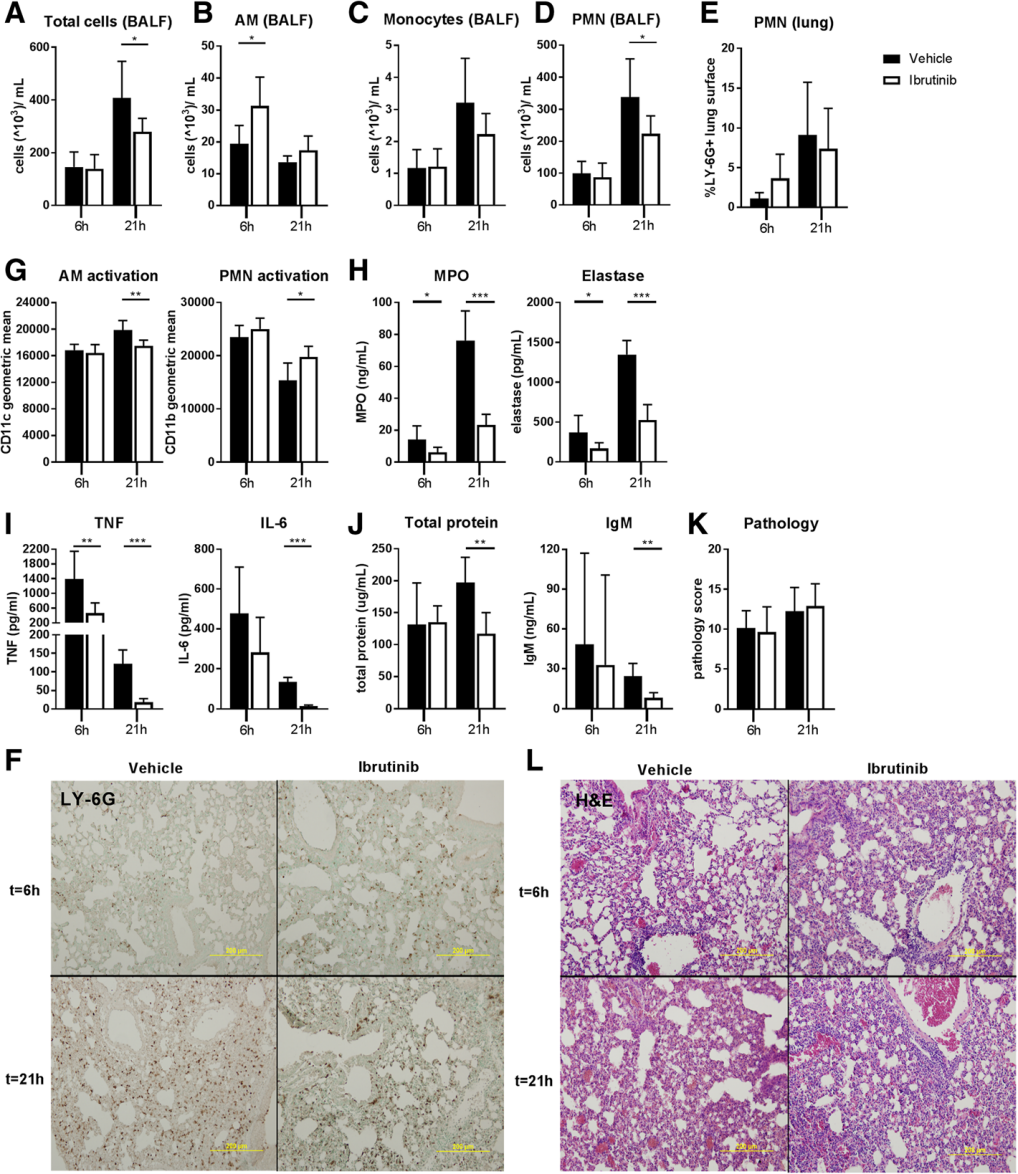
Ibrutinib reduces inflammatory myeloid cell responses during LTA-induced acute lung inflammation. Mice were treated with vehicle (*n* = 8) or ibrutinib (*n* = 8) via oral gavage and sacrificed 6 and 21 h after intranasal administration of lipoteichoic acid (LTA). Numbers of (**a**) total cells in bronchoalveolar lavage fluid (BALF), (**b**) alveolar macrophages (AM), (**c**) monocytes and (**d**) polymorphonuclear cells (PMN) in BALF are depicted as 10^3^cells/mL. (**e**) Percentage of LY-6G positive lung surface as a measure for total lung PMN numbers and (**f**) representative pictures of LY-6G staining. **g** CD11c expression (geometric mean) on BALF AM and CD11b expression (geometric mean) on BALF PMN. **h** Concentrations of PMN granular proteins myeloperoxidase (MPO) and elastase in BALF. **i** Tumor necrosis factor (TNF) and interleukin-6 (IL-6) concentrations in BALF. **j** Concentrations of total protein and immunoglobulin (Ig) M in BALF as a measure for plasma leakage into the lung. **k** Pathology score and **l** representative hematoxylin and eosin (**h**&**e**) pictures. Data are represented as mean and SD. *p < 0.05, **p < 0.01, ***p < 0.001 (Mann-Whitney U-test). Data represent results from a single experiment

To determine whether ibrutinib was capable of diminishing LTA-induced lung inflammation, we treated mice orally with ibrutinib or vehicle and analyzed inflammatory responses 6 and 21 h after intranasal LTA administration. The strategy for in vivo ibrutinib treatment was based on calcium flux analyses of spleen B cells isolated from ibrutinib or vehicle-treated mice (Additional file 2: Figure S1). Ibrutinib or vehicle treatment was commenced 3 h prior to LTA instillation and repeated 12 h after the first treatment for analysis of lung inflammation at 21 h. After 6 h of LTA inflammation, no difference in pulmonary total cell counts in BALF was detected between groups. At 21 h, however, ibrutinib treatment caused a significant reduction in total BALF cells (*p* = 0.04) (Fig. 2a). Flow cytometric analysis of cell types in BALF showed that at 6 h more alveolar macrophages (AM) were retrieved from the ibrutinib treated group as compared to the vehicle treated group (*p* = 0.01), whereas monocyte and PMN numbers were similar (Fig. 2b-d and Additional file 3: Figure S2A). At 21 h of LTA inflammation, AM and monocyte counts were similar between groups, but PMN numbers were reduced by ibrutinib treatment (*p* = 0.04) (Fig. 2b-d). Analysis of PMN numbers in lung tissue by immunohistochemistry showed no differences between groups at 6 and 21 h after LTA inoculation (Fig. 2e-f).

To assess whether ibrutinib altered activation of AM and PMN in the lung, flow cytometric analysis of CD11c and CD11b, respectively, was done. At 6 h, no differences were found in CD11c and CD11b expression, but at 21 h expression of CD11c on AM was significantly reduced in ibrutinib treated mice (*p* = 0.001) (Fig. 2g and Additional file 4: Figure S3A), whereas CD11b expression on PMN was increased (*p* = 0.01) (Fig. 2g and Additional file 4: Figure S3A). To determine whether ibrutinib affected levels of PMN derived factors in BALF, we measured myeloperoxidase (MPO) and elastase. Ibrutinib treatment lowered MPO (*p* = 0.02 and *p* = 0.0003 respectively) and elastase (*p* = 0,04 and *p* = 0.0002 respectively) concentrations at 6 and 21 h (Fig. 2h). A clear effect of ibrutinib was also found on cytokine levels in BALF. Ibrutinib treatment significantly lowered TNF levels at 6 and 21 h (*p* = 0.002 and *p* = 0.0002 respectively) and interleukin (IL)-6 levels at 21 h (*p* = 0.0002) (Fig. 2I). Levels of the chemokines CXCL1 and CXCL2, however, were not altered (Additional file 5: Table S1).

To determine if vascular leakage was influenced by ibrutinib treatment, we measured BALF total protein and IgM levels. Both total protein and IgM levels were not different at 6 h, but were significantly reduced in the ibrutinib treated group at 21 h (*p* = 0.002 and *p* = 0.001 respectively) (Fig. 2j). Ibrutinib treatment, however, did not influence lung pathology (Fig. 2k-l). Together these results indicate that ibrutinib is capable of inhibiting several critical myeloid cell responses during LTA-induced acute lung inflammation.

### Ibrutinib reduces inflammatory myeloid cell responses in the lung during antibiotic-treated pneumococcal pneumonia

**Fig. 3:**
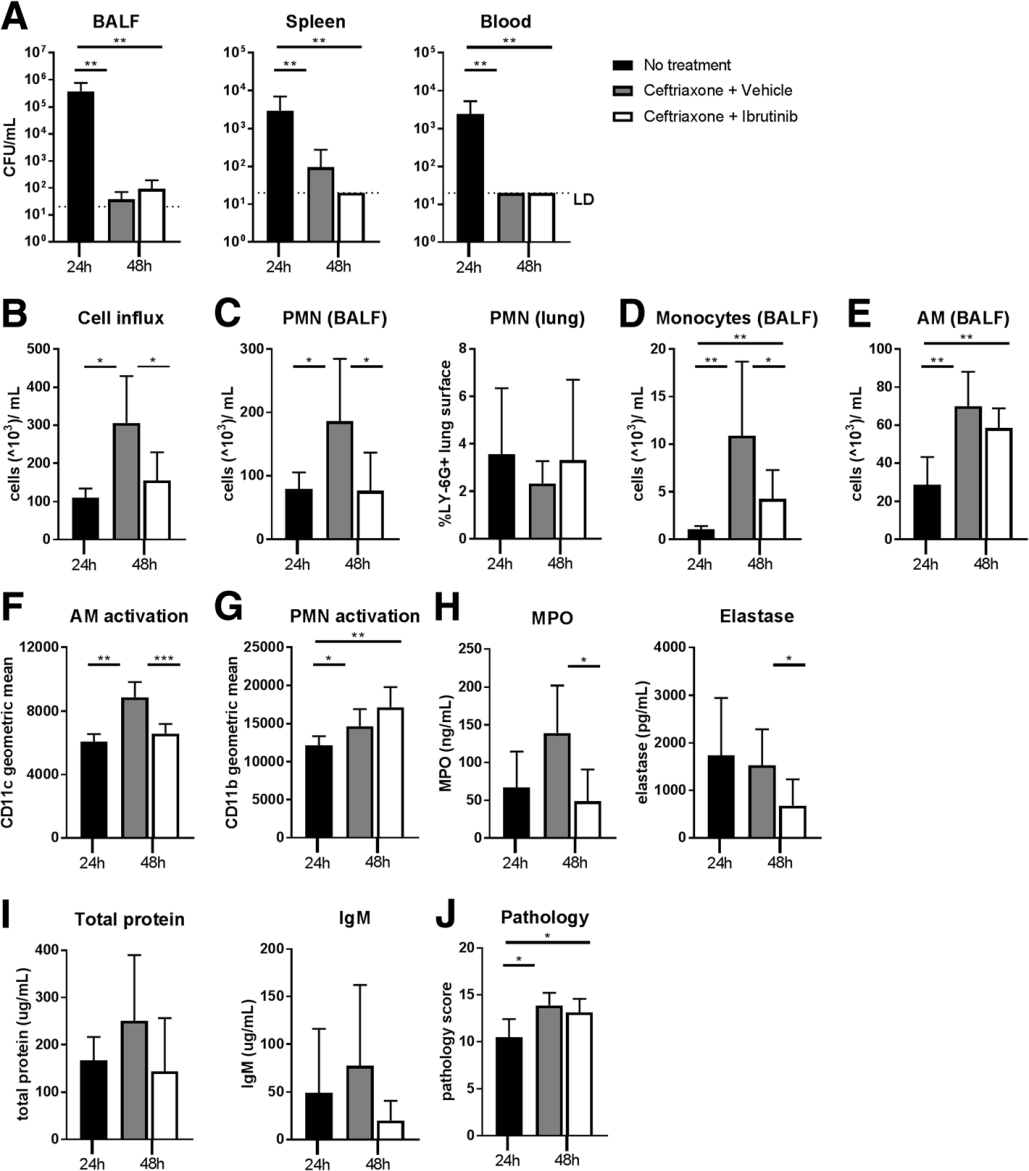
Ibrutinib reduces inflammatory myeloid cell responses in the lung during ceftriaxone-treated pneumococcal pneumonia. Mice were infected intranasally with *S.pneumoniae* bacteria and treated 24 h later with ceftriaxone. Concomitant with ceftriaxone and 12 h later, vehicle or ibrutinib was given and mice were sacrificed 48 h after induction of infection. Mice sacrificed at 24 h (*n* = 4) after inoculation with *S. pneumoniae* without additional treatment served as control. **a** Colony forming units (CFU) in bronchoalveolar lavage fluid (BALF), spleen and blood. **b** Total cells. (**c**, left panel) polymorphonuclear cells (PMN), (**d**) monocytes and (**e**) alveolar macrophags (AM) in BALF depicted as 10^3^cells/mL. (**c**, right panel) percentage of LY-6G positive lung surface as a measure for total lung PMN numbers. **f** CD11c expression (geometric mean) on BALF AM. **g** CD11b expression (geometric mean) on BALF PMN. **h** concentrations myeloperoxidase (MPO) and elastase in BALF. **i** Concentrations of total protein and immunoglobulin (Ig) M in BALF as a measure for plasma leakage into the lung. **j** Lung pathology score. Data are represented as mean and SD. *p < 0.05, **p < 0.01, ***p < 0.001 (Kruskal-Wallis 1-way analysis of variance test followed by Mann-Whitney U-test). Data represent results from a single experiment

Next, we investigated the effect of ibrutinib on an already ongoing inflammatory response in the lung in the established model of pneumococcal pneumonia (Hoogendijk et al., 2012; Rijneveld et al., 2003). Mice were infected intranasally with highly virulent serotype 3 *S.pneumoniae* bacteria and treated 24 h later with ceftriaxone. Concomitant with ceftriaxone and 12 h later, vehicle or ibrutinib was given and mice were sacrificed 48 h after bacterial inoculation. Mice sacrificed at 24 h after bacterial inoculation without additional treatment served as control (*n* = 4), to assess inflammatory parameters at the start of treatment. Ceftriaxone treatment significantly reduced CFU in BALF, spleen and blood compared to the 24 h control group (vehicle or ibrutinib vs control all *p* < 0.01) (Fig. 3a). Ibrutinib treatment did not alter bacterial numbers compared to vehicle treatment.

Analysis of BALF cells (Additional file 3: Figure S2B) showed that the number of cells in vehicle treated mice at 48 h increased as compared to the 24 h controls (*p* = 0.02), indicative of ongoing inflammation despite bacterial eradication. Total cell numbers in the ibrutinib treatment group, however, were markedly reduced as compared to the vehicle treatment group (*p* < 0.03) and similar to the untreated group (Fig. 3b). In vehicle treated mice, PMN numbers increased as compared to the 24 h time point (*p* = 0.04). Ibrutinib treated mice had lower PMN numbers compared to the vehicle treatment group (*p* = 0.02)(Fig. 3c). Analysis of total PMN numbers in the lung, as determined by the percentage of LY-6G positive lung surface, showed no difference between the three groups (Fig. 3c and Additional file 6: Figure S4A). Monocyte numbers in BALF also increased in the vehicle treatment group as compared to the 24 h controls (*p* < 0.004). Ibrutinib treatment largely reduced the influx of monocytes as compared to vehicle treatment (*p* = 0.03), but monocyte numbers were slightly higher as compared to the 24 h controls (*p* = 0.008) (Fig. 3d). AM numbers were increased at 48 h as compared to 24 h (*p* < 0.01 for both treatment groups vs controls), but no difference was found between ibrutinib and vehicle treatment (Fig. 3e).

Flow cytometry analysis of CD11c on AM and CD11b on PMN was employed to assess activation of these cell types in the lung. The vehicle treatment group showed a significant induction in CD11c expression on AM compared to 24 h time point (*p* = 0.004). Ibrutinib treatment, however, abrogated the increase in CD11c expression on AM (*p* = 0.0002) (Fig. 3f and Additional file 4: Figure S3B). PMN CD11b expression was significantly induced in both vehicle and ibrutinib treated mice as compared to the 24 h controls (*p* < 0.05 for both treatment groups vs controls). CD11b expression did not differ between vehicle and ibrutinib treated mice (Fig. 3g and Additional file 4: Figure S3B). Analysis of PMN derived factor concentrations showed that both MPO and elastase levels in BALF were strongly reduced by ibrutinib treatment (*p* = 0.01 and *p* = 0.02 respectively)(Fig. 3h).

In line with bacterial eradication, ceftriaxone abrogated TNF release in BALF (Additional file 7: Table S2). Ibrutinib treatment did not influence cytokine and chemokine release in BALF as compared to vehicle treatment (Additional file 7: Table S2).

Analysis of plasma leakage into the lung as represented by BALF protein and IgM levels showed no significant changes, but ibrutinib treated mice did show a trend towards decreased protein and IgM levels compared to vehicle treated mice (*p* = 0.06 and *p* = 0.18, respectively) (Fig. 3i). Lung pathology slightly increased from 24 h to 48 h after infection (*p* = 0.01 for both vehicle or ibrutinib vs control)(Fig. 3j and Additional file 6: Figure S4B), but was not different between the ibrutinib treatment group and the vehicle treatment group. These results reveal that ibrutinib is able to reduce specific aspects of augmenting acute lung inflammation during antibiotic-treated pneumococcal pneumonia.

### Ibrutinib reduces systemic cell activation during antibiotic-treated pneumococcal pneumonia

**Fig. 4:**
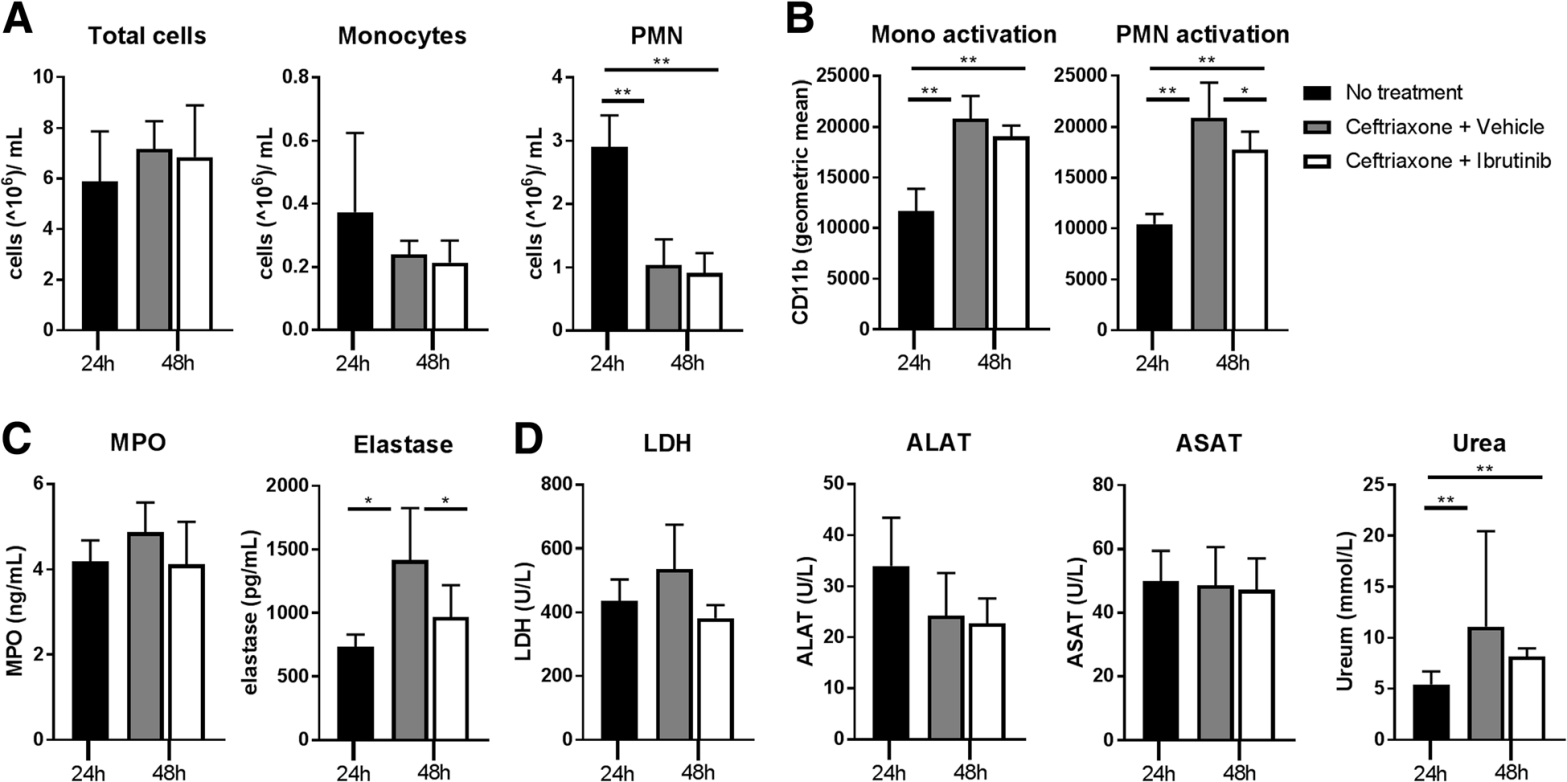
Ibrutinib reduces systemic cell activation during ceftriaxone-treated pneumococcal pneumonia. Mice were infected intranasally with *S.pneumoniae* bacteria and treated 24 h later with ceftriaxone. Concomitant with ceftriaxone and 12 h later, vehicle or ibrutinib was given and mice were sacrificed 48 h after induction of infection. Mice sacrificed at 24 h after inoculation with *S. pneumoniae* without additional treatment served as control. **a** total cells, monocytes and polymorphonuclear cells (PMN) in blood depicted as 10^6^cells/mL. **b** Expression (geometric mean) of CD11b on blood monocytes and PMN. **c** Concentrations of myeloperoxidase (MPO) and elastase in plasma. **d** Concentration of lactate dehydrogenase (LDH), alanine aminotransferase (ALAT), aspartate aminotransferase (ASAT) and urea in plasma. Data are represented as mean and SD. *p < 0.05, **p < 0.01 ((Kruskal-Wallis 1-way analysis of variance test followed by Mann-Whitney U-test). Data represent results from a single experiment

Since *S. pneumoniae* infected mice were bacteremic at the time ibrutinib treatment was commenced (Fig. 3a), we also analyzed the effect of ibrutinib on systemic inflammatory responses during antibiotic-treated pneumococcal pneumonia. Measurement of blood total leukocyte and monocyte numbers at 24 h after infection and in vehicle or ibrutinib treated mice at 48 h after infection showed no differences between groups (Fig. 4a). The number of blood PMN in both treatment groups was decreased as compared to the 24 h time point (both *p* < 0.01)(Fig. 4a and Additional file 3: Figure S2C).

To assess activation of circulating monocyte and PMN, we analyzed expression of CD11b. On monocytes, expression of CD11b was increased in vehicle and ibrutinib treated mice as compared to 24 h controls (*p* = 0.004 for both vehicle or ibrutinib vs control) (Fig. 4b and Additional file 4: Figure S3C). Ibrutinib treatment resulted in a trend towards decreased CD11b expression as compared to vehicle treatment (*p* = 0.08). On PMN, CD11b expression was significantly increased in vehicle treated mice as compared to 24 h controls (*p* = 0.004), but ibrutinib decreased CD11b expression as compared to vehicle (*p* = 0.04) (Fig. 4b and Additional file 4: Figure S3C). To analyze PMN activation further, we determined plasma MPO and elastase levels (Fig. 4c). MPO did not show significant changes between 24 and 48 h or between treatment groups (Fig. 4c). Plasma elastase concentrations were increased in vehicle treated mice at 48 h as compared to the 24 h controls (*p* = 0.02). Ibrutinib treatment strongly reduced elastase concentrations as compared to vehicle treatment (*p* = 0.02) (Fig. 4c).

To assess whether ibrutinib influenced organ damage, we measured plasma levels of lactate dehydrogenase (LDH) as a marker of general cell death, alanine aminotransferase (ALAT) and aspartate aminotransferase (ASAT) as markers of liver damage and urea as marker for kidney injury. Only plasma urea levels increased at 48 h in both treatment groups (*p* = 0.006 for both treatment groups vs control) as compared to the 24 h control group (Fig. 4d). Ibrutinib treatment did not significantly reduce LDH, ALAT, ASAT or urea as compared to vehicle treatment, although a trend to lower plasma LDH levels was noted (*p* = 0.1). Assessment of the effect of ibrutinib on plasma cytokines was inconclusive, as ceftriaxone treatment decreased the levels of TNF, IL-6, CCL2 and interferon-γ (IFNγ) below the limit of detection (Additional file 7: Table S2).

Together, these data suggest that ibrutinib reduces activation of circulating neutrophils during ceftriaxone-treated pneumococcal pneumonia.

## Discussion

Lung inflammation is crucial for host defense against invading pathogens which have entered the lower airways. Overwhelming lung inflammation, however, is unwanted as this may hamper lung function and has detrimental effects on the host (Angus & van der Poll, 2013; van der Poll et al., 2017; Muller-Redetzky et al., 2015). In the present study, we investigated the effect of the Btk inhibitor ibrutinib on acute lung inflammatory responses induced by LTA and by highly virulent serotype 3 *S. pneumoniae* in a clinically relevant setting, i.e. in the context of postponed treatment supplementing antibiotic therapy. In a prophylactic approach, ibrutinib treatment diminished several aspects of LTA-induced lung inflammation, including cytokine release, AM activation and PMN influx into the bronchoalveolar space, which most likely caused the decrease in MPO and elastase levels, and plasma leakage into the lung. Postponed treatment with ibrutinib also reduced inflammatory myeloid cell responses in the lung and circulation during ongoing pneumococcal pneumonia without affecting bacterial clearance. In this model, established lung inflammation was present at the start of treatment and further progressed despite bacterial eradication by ceftriaxone. In this setting, ibrutinib reduced AM activation, reduced systemic PMN activation and substantially diminished monocyte and PMN migration into the bronchoalveolar space. Also during pneumococcal pneumonia, the decreased PMN influx most likely caused the decreased MPO and elastase levels. The effect of ibrutinib on acute inflammatory responses during ceftriaxone-treated pneumococcal pneumonia was not as pronounced as in LTA-induced lung inflammation likely because ibrutinib treatment was started 24 h after induction of infection, thus well after the initiation of inflammatory processes in the lung and activation of Btk expressing cells. Furthermore, Ibrutinib treatment did not alter the number of PMN in the lung parenchyma and total lung pathology evoked by LTA or *S. pneumoniae* and had no effect on levels of plasma markers for organ injury in the later model, presumably because the majority of resident lung l cells do not express Btk but contribute significantly to these aspects of lung inflammation (Dudek et al., 2016). Nevertheless, these findings indicate that ibrutinib has the capacity to ameliorate several aspects of the acute inflammatory response in the lung during ongoing pneumococcal pneumonia. Whether ibrutinib has potential as adjuvant treatment to antibiotics in patients who are at risk of developing acute respiratory distress syndrome or sepsis requires further investigation.

Opposite to the inhibition of various inflammatory responses in the lung, ibrutinib enhanced certain inflammatory parameters, including AM numbers and CD11b expression on PMN after LTA administration. Although total PMN numbers in the bronchoalveolar space after LTA administration were decreased upon ibrutinib treatment, their increased CD11b expression suggests that highly activated blood PMN were still capable to migrate into the airways. Moreover, the higher number of AM in BALF of ibrutinib treated mice early after LTA administration may be explained by decreased adherence of ibrutinib treated AM to the lung epithelium, as observed previously in mice with impaired integrin expression (Anas et al., 2016). Further experiments are required to determine whether ibrutinib alters integrin expression on AM and to assess whether the observed modest changes in CD11b and CD11c expression produced by ibrutinib are biologically significant.

The beneficial effect of ibrutinib on acute inflammatory responses in the lung may result from direct interference of receptor signaling in AM and PMN. TLR2 is essential for responsiveness of AM to LTA and *S. pneumoniae* (Knapp et al., 2004; Knapp et al., 2008) and required for PMN responses to LTA (Lotz et al., 2004). Furthermore, TLR2 is an important receptor for development of both LTA-induced and *S. pneumoniae*-induced lung inflammation (Knapp et al., 2004; Knapp et al., 2008). Previously, it has been shown that Btk regulates LTA-induced TLR2 responses in macrophages (Liljeroos et al., 2007). In line with these findings, we found that Btk controls LTA and *S. pneumoniae*-induced macrophage and PMN activation in vitro and in vivo. The amelioration of inflammatory responses in the lung during pneumococcal pneumonia by ibrutinib may also result from inhibition of other Btk-dependent receptors that regulated *S. pneumoniae*-induced lung inflammation (Hommes et al., 2014; Branger et al., 2004; Albiger et al., 2007; van Lieshout et al., 2018), including TLR4, TLR9, TREM-1 and NLRP3 (Weber et al., 2017; Ito et al., 2015; Ormsby et al., 2011). Additionally, the amelioration of acute lung inflammatory responses by ibrutinib may result from direct inhibition of Btk downstream PSGL-1, CD44 and G-protein receptors in PMN (Yago et al., 2010; Mueller et al., 2010; Volmering et al., 2016), which are essential for PMN migration (Hyun & Hong, 2017). Of note, besides Btk, ibrutinib may target other Tec and Src family kinases (Honigberg et al., 2010), but none of these have been described to affect LTA or *S. pneumoniae*-induced lung inflammation to our knowledge.

Besides directly affecting PMN responses, the anti-inflammatory effect of ibrutinib in the lung compartment may also result from decreased vascular cell activation caused by reduced TNF production. TNF is important for upregulation of adhesion molecules on vascular endothelial cells, and subsequent PMN adherence and migration (Hyun & Hong, 2017). The finding that ibrutinib reduced vascular leakage during LTA-induced lung inflammation supports the hypothesis that ibrutinib may also act indirectly on cells, as endothelial cells do not express Btk. However, it is unlikely that the amelioration of acute inflammatory reponses in the lung by ibrutinib resulted solely from reduced TNF production, since we previously found that anti-TNF therapy impaired bacterial clearance during ceftriaxone-treated pneumococcal pneumonia and aggravated pathology (Rijneveld et al., 2003).

The results of the present study are in line with previously published work showing that inhibition of Btk protected against acute lung injury evoked by polymicrobial sepsis (Zhou et al., 2014), the combination of lipopolysaccharide and immune complexes (Krupa et al., 2014) or the combination of trauma and hemorrhagic shock (Liu et al., 2017). Recently, it was described that intranasal administration of ibrutinib ameliorated survival and decreased lung pathology, PMN influx, neutrophil extracellular trap formation, plasma leakage and cytokine production after influenza infection (Florence et al., 2018). Although these findings suggest that ibrutinib has potency to ameliorate specific aspects of lung inflammation, further investigations have to be done to assess the effectiveness, persistence and safety of this irreversible Btk inhibitor in acute myeloid cell-mediated inflammation.

## Conclusions

The results of the current study reveal that ibrutinib inhibits myeloid cell responses during lung inflammation evoked in mice by LTA and antibiotic-treated pneumococcal pneumonia. Our findings suggest that ibrutinib has the potential to inhibit several aspects of ongoing lung inflammation in acute infectious settings by diminishing myeloid cell activation and migration.

## Additional files

Additional file 1: Supplemental methods (DOC 71 kb)

Additional file 2: **Figure S1.** Ibrutinib inhibits calcium flux of splenic B cells in vivo. Maximum ratio of bound/free indo-1 AM during anti-IgM induced calcium flux experiments on isolated splenic B cells from CD19-BTK and Btk^−/−^ mice treated with vehicle or ibrutinib 3 and 12 h previously (*n* = 4 for the vehicle group, *n* = 2 for the other groups). CD19-BTK mice, Btk^−/−^ mice with transgenic expression of human Btk under the CD19 promotor, were used for their robust calcium flux (Kil et al., 2012). Btk^−/−^ mice were included as a control. Calcium flux was inhibited completely at 3 h after ibrutinib treatment since at this time point the calcium flux was similar to Btk^−/−^ splenic B cells treated with ibrutinib. 12 h after ibrutinib treatment calcium flux was partially restored. Data are represented means with SD or representative histograms are shown. (DOC 273 kb)

Additional file 3: **Figure S2.** Flow cytometric gating strategy to determine the percentage of cell subsets in BALF and blood. (a) Gating strategy to determine the percentage of alveolar macrophages (AMs), monocytes (monos) and polymorphonuclear cells (PMN) in BALF after intranasal LTA administration. (b) Gating strategy to determine the percentage of AMs, monos and PMN in BALF after intranasal *S.pneumoniae* administration. (c) Gating strategy to determine the percentage of monos and PMN in blood. (DOC 1368 kb)

Additional file 4: **Figure S3.** Representative histograms of markers for cell activation. (a) Representative histograms of CD11c expression on alveolar macrophages (AMs) and CD11b expression on polymorphonuclear cells (PMN) in BALF after intranasal LTA administration. (b) Representative histograms of CD11c expression on alveolar macrophages (AMs) and CD11b expression on polymorphonuclear cells (PMN) in BALF after intranasal *S.pneumoniae* administration. (c) Representative histograms of CD11b expression on monocytes (mono) and polymorphonuclear cells (PMN) in blood after intranasal *S.pneumoniae* administration. (DOC 257 kb)

Additional file 5: **Table S1.** BALF chemokine levels 6 and 21 h after intranasal LTA administration in vehicle and ibrutinib treated mice. (DOC 40 kb)

Additional file 6: **Figure S4.** Ibrutinib does not alter lung pathology or PMN influx into the lung during pneumococcal pneumonia. Mice were infected intranasally with *S.pneumoniae* and treated 24 h later with ceftriaxone. Concomitant with ceftriaxone and 12 h later, vehicle or ibrutinib treatment was given and mice were sacrificed 48 h after induction of infection. Mice sacrificed at 24 h after inoculation with *S. pneumoniae* without additional treatment served as control. Representative pictures of Ly-6G (a) and H&E (b) of mice 24 h (left panel) or 48 h (middle and right panels) after intranasal infection with *S. pneumoniae* are shown. (DOC 5684 kb)

Additional file 7: **Table S2.** BALF and plasma cytokine and chemokine levels in a clinical model of *S.pneumoniae* infection. (DOC 44 kb)

## Abbrevations

ALAT: Alanine aminotransferase
AM: Alveolar macrophages
ASAT: Aspartate aminotransferase
BALF: Bronchoalveolar lavage fluid
Btk: Bruton’s tyrosine kinase
CCL: C-C motif ligand
CFU: Colony forming units
CXCL: C-X-C motif ligand
IFNγ: Interferon-γ
IL: Interleukin
LDH: Lactate dehydrogenase
LTA: Lipoteichoic acid
MPO: Myeloperoxidase
NLRP3: Nod-like receptor family pyrin domain containing 3
PMN: Polymorphonuclear cell
*S. pneumoniae*: *Streptococcus pneumoniae*
TLR: Toll-like receptor
TNF: Tumor necrosis factor
TREM: Triggering receptor expressed on myeloid cells
