# Supplementary material for: Btk inhibitor ibrutinib reduces inflammatory myeloid cell responses in the lung during murine pneumococcal pneumonia
Source: Mol Med. 2019 Jan 15;25:3. doi: 10.1186/s10020-018-0069-7 (PMC6332549; doi:10.1186/s10020-018-0069-7)
Supplement: Supplementary file 1 — Supplemental methods (DOC 71 kb) [file 10020_2018_69_MOESM1_ESM.doc]

# METHODS

## Experimental study design

The efficacy and treatment strategy of ibrutinib *in vivo* was determined by analysis of the inhibition of calcium mobilization in spleen B cells. Mice were treated orally with vehicle (5% mannitol, 0.5% gelatin, 7% dimethylsulfoxide (DMSO) in water) or 25 mg/kg body weight of ibrutinib [1] in vehicle and sacrificed 3 or 12 hours later, after which the spleen was isolated. Splenocytes were further processed for flow cytometry.

To study the effect of ibrutinib on lung inflammation, mice (n=8 per group) were treated orally with vehicle or ibrutinib in vehicle (as described above) 3 hours prior to intranasal administration of 100 µg LTA-*S.aureus* (SA) ultrapure (Invivogen, San Diego, CA) in 50 µL phosphate buffered saline (PBS) under isoflurane (Upjohn, Ede, NL) anesthesia [2]. Mice were anesthetized with ketamine and medetomidine and sacrificed by puncture of the vena cava inferior and cervical dislocation at 6 hours after induction of lung inflammation. For analysis of lung inflammation at 21 hours after LTA administration, mice were additionally treated with vehicle or ibrutinib at 9 hours after induction of lung inflammation.

Pneumococcal pneumonia was induced as previously described [2]. Briefly, mice were inoculated intranasally with 2x10^5^ CFU of strain 6303 (serotype 3; American Type Culture Collection, Manassas, VA) in 50uL of PBS during isoflurane anesthesia. At 24 hours after infection, one group of mice (n=4) was sacrificed to obtain control samples and two groups of mice (n=8) were treated intraperitoneally with 20 mg/kg ceftriaxone (Fresenius Kabi, Zeist, the Netherlands) in 200 µL saline of which one group was treated simultaneously with vehicle and the other with ibrutinib in vehicle, as described above. At 36 hours after infection, vehicle or ibrutinib treatment was repeated and at t=48 hours mice were sacrificed.

After sacrifice, bronchoalveolar lavage fluid (BALF), blood and spleen were harvested from all mice and processed as described previously [2].

## Calcium mobilization

For measurement of calcium flux, spleens were crushed through a 100 µm mesh, diluted in Hanks Balanced Salt Solution containing 5% fetal calf serum and lymphocytes were collected using Ficoll paque plus (GE Healthcare, Hoevelaken, Netherlands). Cells were loaded with Indo-1 AM (ThermoFisher Scientific) at 37 ^o^C for 30 minutes and stained with rat anti-mouse B220-FITC (clone RA3-6B2, BD Biosciences, San Jose, CA) at 4 ^o^C. Samples were analyzed using a LSR Fortessa (BD Biosciences). The ratio of calcium bound to free indo-1AM was first measured under steady state for 1 minute, after which 12.5 µg/mL goat F(ab’)_2_ anti-mouse IgM (SouthernBiotech, Birmingham, AL) was added and calcium flux was measured for 4 minutes. Data were analyzed using FlowJo software (Tree Star, Ashland, OR).

*Cell stimulation*

RAW264.7 (ATCC, Manassas, VA) were cultured in Iscove’s Modified Dulbecco’s Medium (Gibco; ThermoFisher Scientific) supplemented with 5% L-glutamine (Sigma-Aldrich, St. Louis, MO), 0.1 units / mL penicillin-streptomycin (Sigma-Aldrich) and 10% fetal calf serum (Hycult, Uden, the Netherlands) as previously described [2]. Cells were plated in a 96 wells flat bottom plate (Greiner Bio-One, [Kremsmünster, Austria)](https://www.google.nl/search?q=Kremsmünster+Oostenrijk&stick=H4sIAAAAAAAAAOPgE-LSz9U3SEvONjGuUgKzjUxLCouMtLSyk63084vSE_MyqxJLMvPzUDhWGamJKYWliUUlqUXFAHA3FnxGAAAA&sa=X&ved=0ahUKEwjDl8-Pjd3ZAhXhB8AKHdwpBJYQmxMImQEoATAR) and allowed to adhere overnight. 30 minutes prior to stimulation cell were incubated with vehicle (medium with 0.1% DMSO) or 2500 nM Ibrutinib in vehicle. Cell were stimulated with 0.0.1, 0.1 and 1 µg/mL LTA-SA ultrapure or UV-killed *S.pneumoniae* 6303 in medium overnight.

Stimulation of RAW264.7 cells for western blot analysis was performed in a 24 well flat bottom plate and cells were allowed to adhere overnight. Cell were stimulated for 5 minutes with 10 µg/mL LTA-SA ultrapure in medium in the presence of vehicle or ibrutinib. After stimulation, cells were washed with ice cold PBS and lysed in ice cold lysis buffer (50 mM TRIS HCl pH 8.0, 150 mM NaCl and 1% Triton-X100 in water) for 30 minutes on ice. The lysate was centrifuged for 10 minutes at 14000g, 4 ^o^C and supernatant was collected and stored at -20 ^o^C for western blot analysis.

Bone marrow neutrophils were isolated as described previously [3] with minor modifications. In brief, bone marrow cells were layered on a discontinuous gradient of Ficoll-Paque (GE Healthcare, Uppsala, Sweden) and Polymorphprep (Axis-Shield, Oslo, Norway) and centrifuged (870xg) for 30 minutes at room temperature. Neutrophils were harvested from the Histopaque/Polymorphprep interface, washed twice with 0.1% BSA in HBSS (without calcium and magnesium) and resuspended in HBSS with 0.5 mM CaCl_2_ and 1 mM MgCl_2_ prior to stimulation. Neutrophils were plated in polypropylene plates, pretreated with vehicle or ibrutinib as described above and stimulated with 10 µg/mL LTA-SA ultrapure or UV-killed *S.pneumoniae* 6303 (100:1 bacterium:cell ratio) in plain RPMI1640 for 1 hour.

*Western blot analysis*

Western blot analysis was performed as described previously [2]. RAW264.7 cells were lysed using lysis buffer, consisting of 50mM TRIS HCl pH8.0, 150mM NaCl and 1% Triton-X100 in water. Blots were incubated with rabbit anti-human phospho-BTK-Y223 antibody (clone EP420Y, Abcam, Cambridge, UK), rabbit anti-Btk (clone D3H5, Cell Signaling Technology, Danvers, MA) and rabbit anti-β-actin (Cell Signaling Technology).

*Flow cytometric cell analysis*

For analysis of cell subsets in blood and BALF (Figure S2a-c) total cell counts were determined with a Coulter cell counter (Beckman Coulter, Fullerton, CA). 45 µL of whole blood was treated with lysis buffer for erythrocytes, consisting of 150 mM NH_4_Cl, 10 mM KHCO_3_, 0.1 mM EDTA pH 7.4 in water for 10 minutes at 4 ^o^C. After lysis of erythrocytes and centrifugation, blood leukocytes and BALF cells were resuspended FACS buffer (5% BSA, 0.35 mM EDTA, 0.01% NaN_3_). Cell staining was performed according to manufacturer’s recommendations using fixable viability dye eFluor 780, rat anti mouse-CD16/CD32 (clone 93), rat anti mouse-CD45 PE-eFluor610 (30-F11), hamster anti-mouse CD11c PerCP-Cy5-5 (clone HL3), rat anti-mouse CD11b PE-Cy7 (clone M1/70), rat anti-mouse Siglec-F Alexa Fluor 647 (clone E50-2440), rat anti-mouse Ly-6G PE (clone 1A8), and hamster anti-mouse CD11c FITC (clone HL3) (all from BD Biosciences); and rat anti-mouse Ly-6G FITC (clone 1A8; Biolegend, San Diego, CA). Analysis of bone marrow derived neutrophils was performed similarly using rat anti-Ly-6G-APC and rat anti-mouse CD11b PE-Cy7. Flow cytometry was performed using a FACSCANTO II (BD Biosciences) and data were analyzed using FlowJo software.

## ELISA and other assays

Murine myeloperoxidase (MPO), elastase, C-X-C motif ligand (CXCL)1, CXCL2, C-C motif ligand (CCL)2, IL-6, Tumor necrosis factor-α (TNF-α) and interferon-y (IFNy) were measured by ELISA (all R&D systems, Minneapolis, MN) or mouse inflammation kit Cytometric Beads Array (BD Biosciences).Total protein was measured using a BCA protein assay Kit (Thermofisher Scientific), IgM was measured using rat anti-mouse IgM (1B4B1) as a capture antibody, mouse IgM (11E10) as a standard and goat anti-mouse IgM-biotin (all from Southernbiotech, Birmingham, AL) as a detection antibody. Plasma aminotransferase (AST), alanine aminotransferase (ALT), urea and lactate dehydrogenase (LDH) were measured using a c702 Roche Diagnostics (Roche Diagnostics BV, Almere, the Netherlands).

## Histopathology and immunohistochemistry

Four micrometer lung sections were stained with hematoxylin and eosin. Slides were coded and lung inflammation (interstitial inflammation, endothelialitis, bronchitis, oedema, pleuritis) and damage was scored by a pathologist blinded for group identity as previously described [2]. For analysis of neutrophil influx in the lung, sections were stained with rat anti-mouse Ly-6G FITC (1A8, 127605, BioLegend) as previously described [2]. Immunohistochemical staining’s were quantified by digital image analysis. Slides were scanned with the Philips intelliSight Ultra FastScanner 1.6RA (Philips digital pathology solutions, Best, The Netherlands) and the amount of immune-positivity was measured as percentage of the total lung surface using Image-Pro Premier (Media Cybernatics,Rockville, MD).

# REFERENCES

1. Kil LP, de Bruijn MJW, van Nimwegen M, et al. Btk levels set the threshold for B-cell activation and negative selection of autoreactive B cells in mice. Blood. 2012;119:3744-3756.

2. Hoogendijk AJ, Roelofs JJ, Duitman J, et al. R-roscovitine reduces lung inflammation induced by lipoteichoic acid and Streptococcus pneumoniae. Mol Med. 2012;18:1086-1095.

3. Swamydas M, Luo Y, Dorf ME, Lionakis MS. Isolation of Mouse Neutrophils. Curr Protoc Immunol. 2015;110:3.20.21-23.20.15.
