# Supplementary material for: Btk inhibitor ibrutinib reduces inflammatory myeloid cell responses in the lung during murine pneumococcal pneumonia
Source: Mol Med. 2019 Jan 15;25:3. doi: 10.1186/s10020-018-0069-7 (PMC6332549; doi:10.1186/s10020-018-0069-7)
Supplement: Supplementary file 2 — Figure S1. Ibrutinib inhibits calcium flux of splenic B cells in vivo. Maximum ratio of bound/free indo-1 AM during anti-IgM induced calcium flux experiments on isolated splenic B cells from CD19-BTK and Btk−/− mice treated with vehicle or ibrutinib 3 and 12 h previously (n = 4 for the vehicle group, n = 2 for the other groups). CD19-BTK mice, Btk−/− mice with transgenic expression of human Btk under the CD19 promotor, were used for their robust calcium flux (Kil et al., 2012). Btk−/− mice were included as a control. Calcium flux was inhibited completely at 3 h after ibrutinib treatment since at this time point the calcium flux was similar to Btk−/− splenic B cells treated with ibrutinib. 12 h after ibrutinib treatment calcium flux was partially restored. Data are represented means with SD or representative histograms are shown. (DOC 273 kb) [file 10020_2018_69_MOESM2_ESM.doc]

**
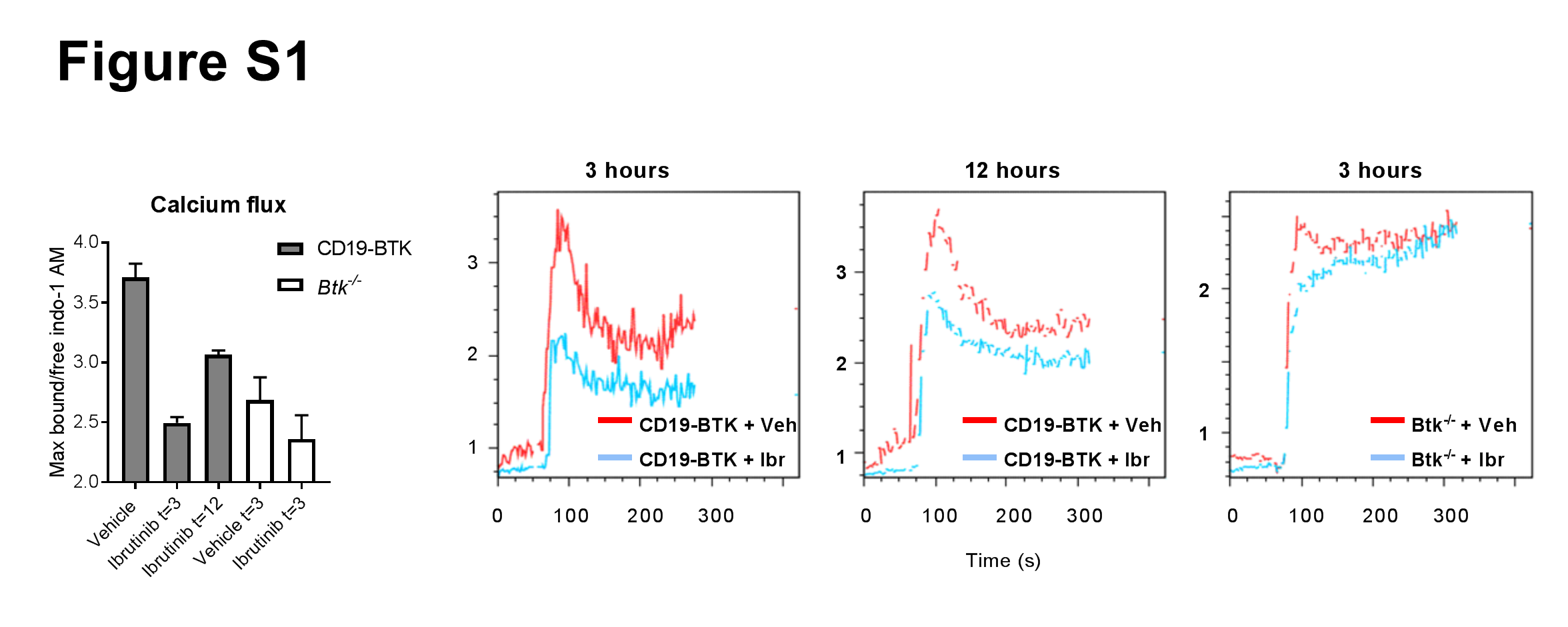
Figure S1 Ibrutinib inhibits calcium flux of splenic B cells in vivo**

Maximum ratio of bound/free indo-1 AM during anti-IgM induced calcium flux experiments on isolated splenic B cells from CD19-BTK and Btk^-/-^ mice treated with vehicle or ibrutinib 3 and 12 hours previously (n=4 for the vehicle group, n=2 for the other groups). CD19-BTK mice, Btk^-/-^ mice with transgenic expression of human Btk under the CD19 promotor, were used for their robust calcium flux [1]. Btk^-/-^ mice were included as a control. Calcium flux was inhibited completely at 3 hours after ibrutinib treatment since at this time point the calcium flux was similar to Btk^-/-^ splenic B cells treated with ibrutinib. 12 hours after ibrutinib treatment calcium flux was partially restored.

Data are represented means with SD or representative histograms are shown.

# REFERENCES

1. Kil LP, de Bruijn MJW, van Nimwegen M, et al. Btk levels set the threshold for B-cell activation and negative selection of autoreactive B cells in mice. Blood. 2012;119:3744-3756.
