# Supplementary material for: Btk inhibitor ibrutinib reduces inflammatory myeloid cell responses in the lung during murine pneumococcal pneumonia
Source: Mol Med. 2019 Jan 15;25:3. doi: 10.1186/s10020-018-0069-7 (PMC6332549; doi:10.1186/s10020-018-0069-7)
Supplement: Supplementary file 3 — Figure S2. Flow cytometric gating strategy to determine the percentage of cell subsets in BALF and blood. (a) Gating strategy to determine the percentage of alveolar macrophages (AMs), monocytes (monos) and polymorphonuclear cells (PMN) in BALF after intranasal LTA administration. (b) Gating strategy to determine the percentage of AMs, monos and PMN in BALF after intranasal S.pneumoniae administration. (c) Gating strategy to determine the percentage of monos and PMN in blood. (DOC 1368 kb) [file 10020_2018_69_MOESM3_ESM.doc]

#
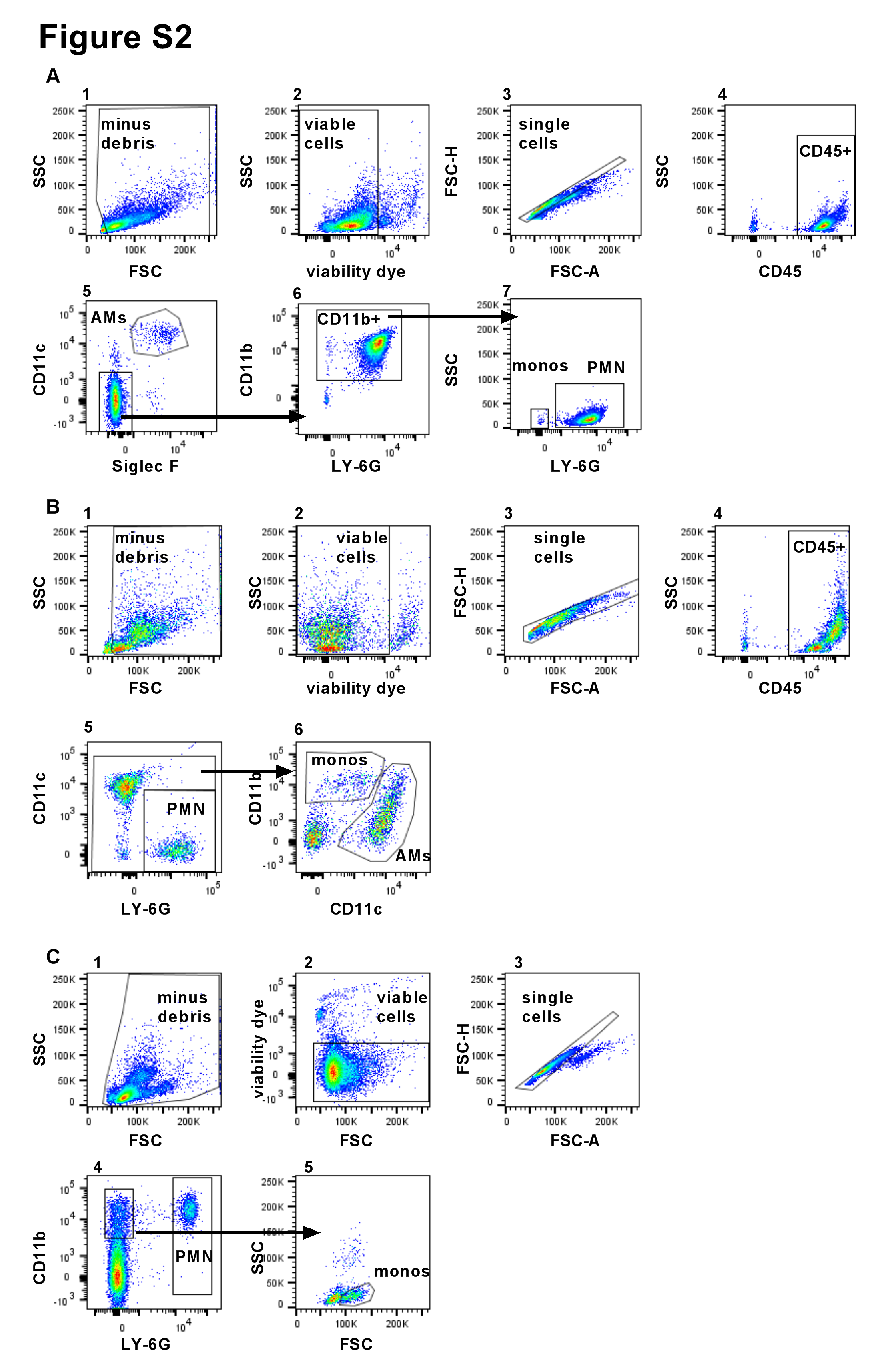
Figure S2 Flow cytometric gating strategy to determine the percentage of cell subsets in BALF and blood.

(a) Gating strategy to determine the percentage of alveolar macrophages (AMs), monocytes (monos) and polymorphonuclear cells (PMN) in BALF after intranasal LTA administration. (b) Gating strategy to determine the percentage of AMs, monos and PMN in BALF after intranasal *S.pneumoniae* administration. (c) Gating strategy to determine the percentage of monos and PMN in blood.
