# Supplementary material for: Btk inhibitor ibrutinib reduces inflammatory myeloid cell responses in the lung during murine pneumococcal pneumonia
Source: Mol Med. 2019 Jan 15;25:3. doi: 10.1186/s10020-018-0069-7 (PMC6332549; doi:10.1186/s10020-018-0069-7)
Supplement: Supplementary file 4 — Figure S3. Representative histograms of markers for cell activation. (a) Representative histograms of CD11c expression on alveolar macrophages (AMs) and CD11b expression on polymorphonuclear cells (PMN) in BALF after intranasal LTA administration. (b) Representative histograms of CD11c expression on alveolar macrophages (AMs) and CD11b expression on polymorphonuclear cells (PMN) in BALF after intranasal S.pneumoniae administration. (c) Representative histograms of CD11b expression on monocytes (mono) and polymorphonuclear cells (PMN) in blood after intranasal S.pneumoniae administration. (DOC 257 kb) [file 10020_2018_69_MOESM4_ESM.doc]

#
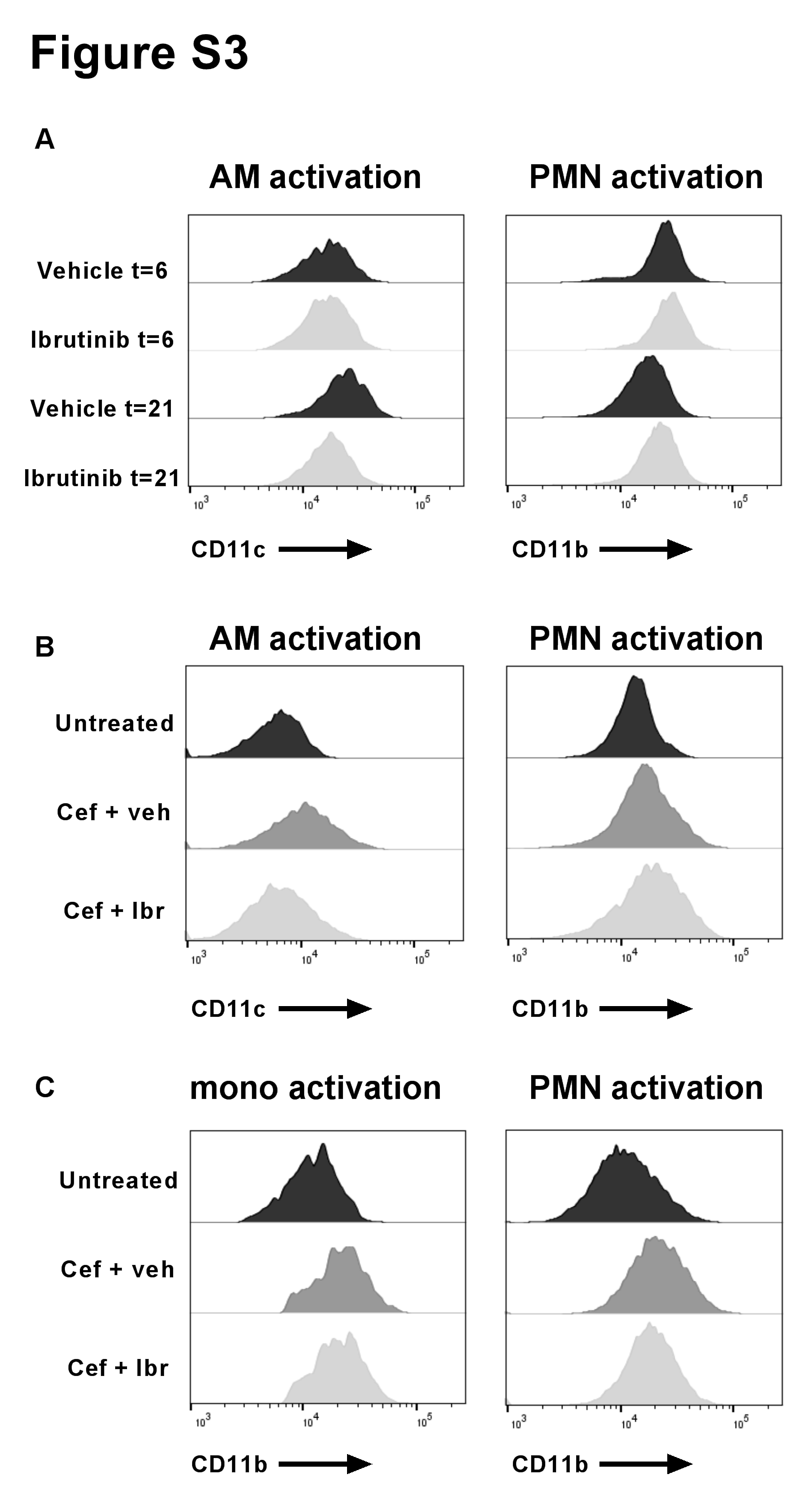


# Figure S3 Representative histograms of markers for cell activation

(a) Representative histograms of CD11c expression on alveolar macrophages (AMs) and CD11b expression on polymorphonuclear cells (PMN) in BALF after intranasal LTA administration. (b) Representative histograms of CD11c expression on alveolar macrophages (AMs) and CD11b expression on polymorphonuclear cells (PMN) in BALF after intranasal *S.pneumoniae* administration. (c) Representative histograms of CD11b expression on monocytes (mono) and polymorphonuclear cells (PMN) in blood after intranasal *S.pneumoniae* administration.
