# Supplementary material for: Btk inhibitor ibrutinib reduces inflammatory myeloid cell responses in the lung during murine pneumococcal pneumonia
Source: Mol Med. 2019 Jan 15;25:3. doi: 10.1186/s10020-018-0069-7 (PMC6332549; doi:10.1186/s10020-018-0069-7)
Supplement: Supplementary file 5 — Table S1. BALF chemokine levels 6 and 21 h after intranasal LTA administration in vehicle and ibrutinib treated mice. (DOC 40 kb) [file 10020_2018_69_MOESM5_ESM.doc]

**Table S1 BALF chemokine levels 6 and 21 hours after intranasal LTA administration in vehicle and ibrutinib treated mice.**

|  | **t=6** | | **t=21** | |
| --- | --- | --- | --- | --- |
| **BALF** | **Vehicle** | **Ibrutinib** | **Vehicle** | **Ibrutinib** |
| CXCL1 | 1175 (602) | 1147 (512) | <LD | <LD |
| CXCL2 | 1572 (328) | 1558 (604) | 800 (86) | 788 (32) |

Ibrutinib or vehicle treatment was commenced 3 hours prior to LTA instillation and repeated 12 hours after the first treatment for analysis of lung inflammation at 21 hours.

Data are mean (SD); LD = limit of detection.
