# Supplementary material for: Btk inhibitor ibrutinib reduces inflammatory myeloid cell responses in the lung during murine pneumococcal pneumonia
Source: Mol Med. 2019 Jan 15;25:3. doi: 10.1186/s10020-018-0069-7 (PMC6332549; doi:10.1186/s10020-018-0069-7)
Supplement: Supplementary file 6 — Figure S4. Ibrutinib does not alter lung pathology or PMN influx into the lung during pneumococcal pneumonia. Mice were infected intranasally with S.pneumoniae and treated 24 h later with ceftriaxone. Concomitant with ceftriaxone and 12 h later, vehicle or ibrutinib treatment was given and mice were sacrificed 48 h after induction of infection. Mice sacrificed at 24 h after inoculation with S. pneumoniae without additional treatment served as control. Representative pictures of Ly-6G (a) and H&E (b) of mice 24 h (left panel) or 48 h (middle and right panels) after intranasal infection with S. pneumoniae are shown. (DOC 5684 kb) [file 10020_2018_69_MOESM6_ESM.doc]

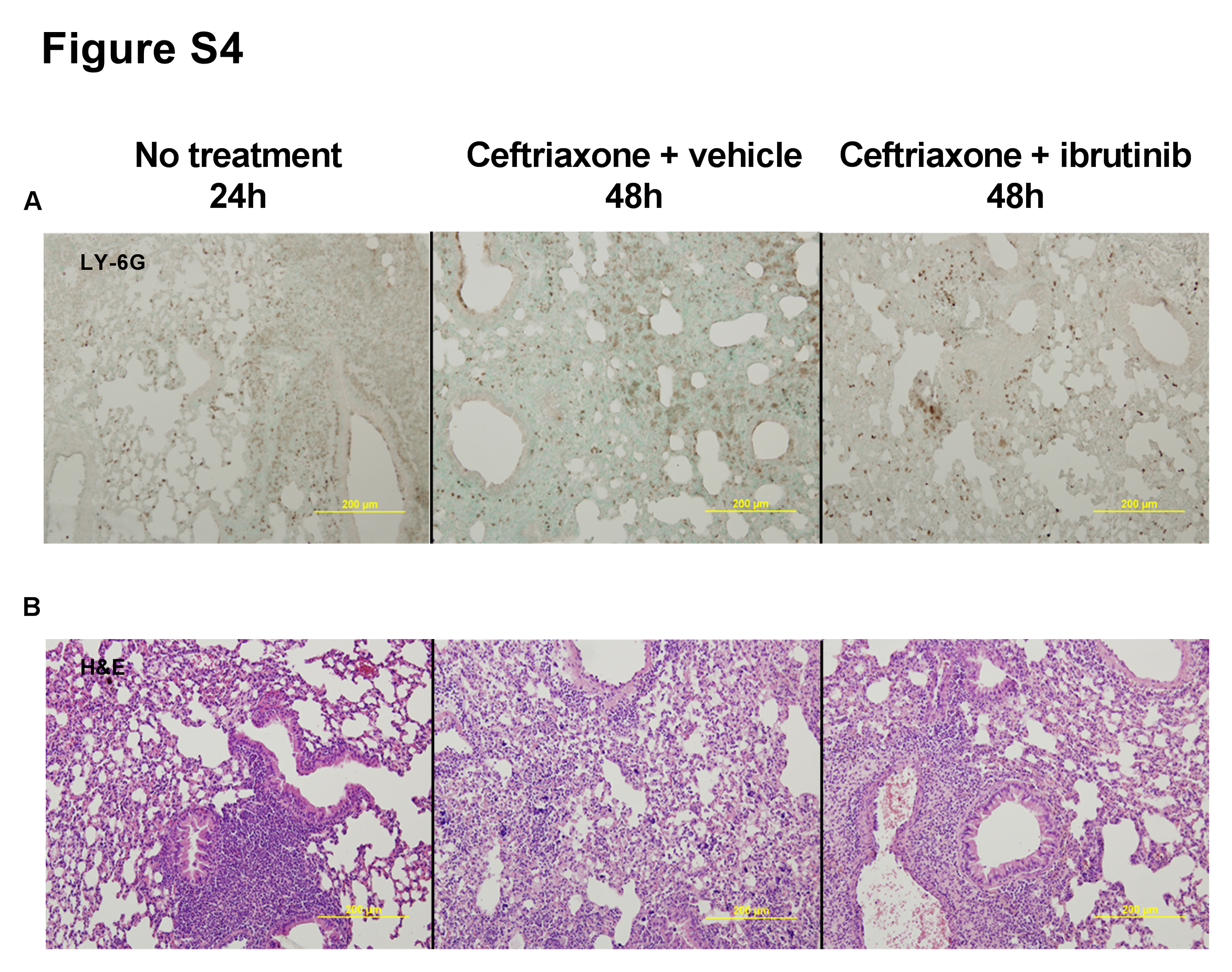


**Figure S4 Ibrutinib does not alter lung pathology or PMN influx into the lung during pneumococcal pneumonia.**

Mice were infected intranasally with *S.pneumoniae* and treated 24 hours later with ceftriaxone. Concomitant with ceftriaxone and 12 hours later, vehicle or ibrutinib treatment was given and mice were sacrificed 48 hours after induction of infection. Mice sacrificed at 24 hours after inoculation with *S. pneumoniae* without additional treatment served as control. Representative pictures of Ly-6G (a) and H&E (b) of mice 24 hours (left panel) or 48 hours (middle and right panels) after intranasal infection with *S. pneumoniae*  are shown.
