# Supplementary material for: Btk inhibitor ibrutinib reduces inflammatory myeloid cell responses in the lung during murine pneumococcal pneumonia
Source: Mol Med. 2019 Jan 15;25:3. doi: 10.1186/s10020-018-0069-7 (PMC6332549; doi:10.1186/s10020-018-0069-7)
Supplement: Supplementary file 7 — Table S2. BALF and plasma cytokine and chemokine levels in a clinical model of S.pneumoniae infection. (DOC 44 kb) [file 10020_2018_69_MOESM7_ESM.doc]

**Table S2 BALF and plasma cytokine and chemokine levels in a clinical model of *S.pneumoniae*** infection

|  | **t=24** | **t=48** | |
| --- | --- | --- | --- |
| **BALF** | **No treatment** | **Cef + Vehicle** | **Cef + Ibrutinib** |
| TNF | 360 (332) | 30 (22)** | 15 (11)** |
| IL-6 | 212 (204) | 68 (90) | 28 (33)* |
| CXCL1 | 715 (714) | 360 (304) | 243 (130) |
| CXCL2 | 936 (187) | 730 (154) | 688 (137)* |
| **Plasma** | **No treatment** | **Cef + Vehicle** | **Cef + Ibrutinib** |
| TNF | 11 (2) | <LD | <LD |
| IL-6 | 104 (33) | <LD | <LD |
| CCL2 | 97 (22) | <LD | <LD |
| IFN-γ | 41 (23) | <LD | <LD |

Mice were infected intranasally with highly virulent serotype 3 *S.pneumoniae* bacteria and treated 24 hours later with ceftriaxone. Concomitant with ceftriaxone and 12 hours later, vehicle or ibrutinib was administered and mice were sacrificed 48 hours after induction of infection. Mice sacrificed at 24 hours after inoculation with *S. pneumoniae* without additional treatment served as control.

Data are mean (SD); LD = limit of detection; Cef= Ceftriaxone*p<0.05;**p<0.01 compared to no treatment
